# Reconstruction of the diapsid ancestral genome permits chromosome evolution tracing in avian and non-avian dinosaurs

**DOI:** 10.1038/s41467-018-04267-9

**Published:** 2018-05-21

**Authors:** Rebecca E. O’Connor, Michael N. Romanov, Lucas G. Kiazim, Paul M. Barrett, Marta Farré, Joana Damas, Malcolm Ferguson-Smith, Nicole Valenzuela, Denis M. Larkin, Darren K. Griffin

**Affiliations:** 1 School of Biosciences, University of Kent, Canterbury, Kent, CT2 7NJ UK; 2 Department of Earth Sciences, Natural History Museum, Cromwell Road, London, SW7 5BD UK; 3 Department of Comparative Biomedical Sciences, Royal Veterinary College, University of London, London, NW1 0TU UK; 4 Department of Veterinary Medicine, Cambridge University, Cambridge, CB3 0ES UK; 5 Department of Ecology, Evolution, and Organismal Biology, Iowa State University, Iowa, IA 50011 USA

## Abstract

Genomic organisation of extinct lineages can be inferred from extant chromosome-level genome assemblies. Here, we apply bioinformatic and molecular cytogenetic approaches to determine the genomic structure of the diapsid common ancestor. We then infer the events that likely occurred along this lineage from theropod dinosaurs through to modern birds. Our results suggest that most elements of a typical ‘avian-like’ karyotype (40 chromosome pairs, including 30 microchromosomes) were in place before the divergence of turtles from birds ~255 mya. This genome organisation therefore predates the emergence of early dinosaurs and pterosaurs and the evolution of flight. Remaining largely unchanged interchromosomally through the dinosaur–theropod route that led to modern birds, intrachromosomal changes nonetheless reveal evolutionary breakpoint regions enriched for genes with ontology terms related to chromatin organisation and transcription. This genomic structure therefore appears highly stable yet contributes to a large degree of phenotypic diversity, as well as underpinning adaptive responses to major environmental disruptions via intrachromosomal repatterning.

## Abstract (web summary)

Ancient diapsids diverged into the lineages leading to turtles and birds over 250 million years ago. Here, the authors use genomic and molecular cytogenetic analyses of modern species to infer the genome structure of the diapsid common ancestor (DCA) and the changes occurring along the lineage to birds through theropod dinosaurs.

## Introduction

In the absence of cellular material and DNA from biological samples of long-extinct, early diverging lineages, data from genome sequence assemblies of extant species can nonetheless facilitate the reconstruction of gross genome structures (karyotypes). This can be achieved provided those assemblies are at, or close to, chromosome level, i.e. one scaffold per chromosome^1^. In a previous study, we analysed (close to) chromosome-level assemblies from six extant birds (and a lizard outgroup) to determine the most likely karyotype of the neornithine ancestor for the macrochromosomes and the neognathe ancestor for the microchromosomes^2^. Recreating the most parsimonious sequence of events that might have led to contemporary genome structures (karyotypes), we determined that chicken (*Gallus gallus*) was the closest karyotypically to the reconstructed ancestral pattern, with zebra finch (*Taeniopygia guttata)* and budgerigar (*Melopsittacus undulatus)* undergoing the most intra- and interchromosomal rearrangements, respectively. In the current study, to reconstruct the most likely karyotype of the diapsid common ancestor (DCA >255 mya), we applied similar approaches, i.e. the multiple-genome rearrangement and analysis (MGRA2) tool. We focussed on the best-quality chromosome-level assemblies of avian and reptilian genomes and a mammalian outgroup. Supplementing bioinformatic data with novel molecular cytogenetic approaches on turtle metaphases, we tested the hypothesis that the typical karyotype seen in neornithine birds underwent few interchromosomal rearrangements since the divergence of turtles from archosaurs (birds and crocodilians) <255 mya. Combining both sets of data, we thence inferred the most parsimonious sequence of events that occurred from the diapsid ancestor, to the archelosaur ancestor^3^, and thence via non-avian theropod dinosaurs to extant birds (see Supplementary Note 1 for divergence times).

Studies of the best-assembled genomes (including chromosome level) also indicate that evolutionary breakpoint regions (EBRs) lie in gene-dense loci, enriched with genes related to lineage-specific biology, transposable elements and other repetitive sequences^4–7^, while sequences that stay together during evolution (homologous synteny blocks, HSBs) are enriched in developmental genes and regulatory elements^7^. While a contribution of random breakage in chromosome evolution^9^ cannot be excluded for all (especially smaller) rearrangements that might have neutral effects on phenotypes, multiple evidence has accumulated to suggest that at least the largest HSBs and some EBRs in animal genomes are maintained non-randomly^4,5,10,11^. Differences in the composition of their DNA features suggest that, although chromosome aberrations in germ cells may indeed occur in regions more prone to breakage (e.g. recombination hotspots or open chromatin areas), those breaks not disturbing essential genes or providing a selective advantage will more likely be fixed in populations, becoming EBRs^4^. In the current study, we investigated gene content of those EBRs and HSBs identified as being involved in the karyotypic changes from the diapsid ancestor to modern birds and identified genes that may indicate adaptive (EBRs) or conserved (HSBs) phenotypic features or those likely to be involved in gross genomic rearrangement.

Here, we analysed data from the genome assemblies of Carolina anole lizard (*Anolis carolinensis)*^6^, chicken (*G. gallus)*^7,12^, mallard (*Anas platyrhynchos*)^8^, zebra finch (*T. guttata*)^13^, and grey short-tailed opossum (*Monodelphis domestica*)^14^. All of these species have robust chromosome-level assembled genomes. Moreover, *M. domestica* has a karyotypic structure thought to resemble the mammalian ancestor most closely^14^. Among other genome assemblies that might have proved useful in our analyses, those generated from alligators and turtles^15,16^ were discounted as they are too fragmented, i.e. not close to chromosome level. Also, near-chromosome-level assemblies for turkey, budgerigar and ostrich were ultimately excluded because our cytogenetic studies in these species (not shown) revealed that the level of fragmentation or misassembles in these genomes had the potential to introduce false breakpoint regions (Supplementary Note 2). Finally, we discounted crocodilians from our molecular analysis, partly because of a relative lack of fluoresence in situ hybridisation (FISH) success of multiple attempts, and partly because all crocodilian species studied have an atypical archelosaur karyotype with no microchromosomes, mostly brought about by fusion^17^.

Our results suggest that most features of a typical ‘avian-like’ karyotype were in place before the divergence of birds and turtles, that the predominant mechanism of change thereafter was intrachromosomal rearrangement and that EBRs were enriched for GO terms associated with chromatin modification and chromosome organisation.

## Results

### Summary of results

**Fig. 1:**
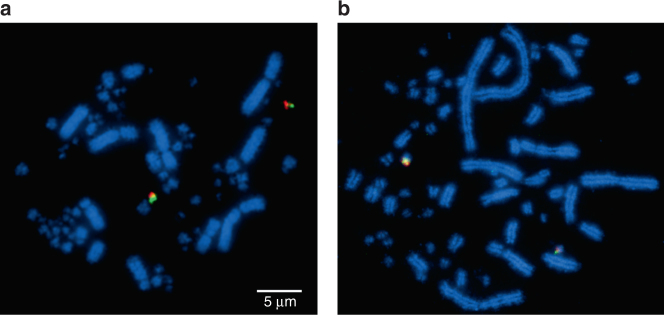
Cross-species hybridisation (zoo-FISH) results. Hybridisation of chicken chromosome 23 sub-telomeric BACs to **a** chicken (2*n* = 78) and **b** turtle (*Apalone spinifera* 2*n* = 66) metaphases. This is an example of how most chromosomes studied in birds and turtles (species examined with the highest diploid number) are precise counterparts of one another. All syntenic chromosomes were of similar sizes and morphologies. All chromosomes are labelled in blue (DAPI) with BAC probes labelled in red (Texas Red) and green (FITC), respectively (where signals overlap a little, a yellow/orange colour is seen). Successful strong hybridisation across large evolutionary distances was one of the technical advances of this project. Scale bar applies to both images

Pairwise sequence alignments permitted visualisation of 397 multispecies HSBs, their orientation in each genome and EBRs between them (listed in Supplementary Data 2). Using MGRA2, we generated 19 contiguous ancestral regions (CARs), roughly correlating to chromosomes, in the most likely ancestral karyotype of the DCA. CAR sizes are given in Supplementary Data 1 and the analysis pipeline is described in Methods. Our analysis of chromosomes from the turtle genome with one of the largest chelonian diploid numbers (2*n*) (spiny soft-shelled turtle (*Apalone spinifera)* (2*n* = 66)) revealed little or no evidence of interchromosomal differences between this species and chicken. That is, chicken-derived fluorescent probes highly selected to hybridise across large evolutionary distances^5^ plus chromosome painting data provided evidence that most chicken chromosomes 1–28 + Z are each represented by a single-turtle counterpart (see Methods and Supplementary Data 3). Successful hybridisation to the chromosomes of red-eared slider (*Trachemys scripta*) (2*n* = 50) revealed a karyotype with microchromosomal homeologues either having fused to macrochromosomes or, more likely, having retained the ancestral state of the DCA. Indeed, one of the main technical advances made in this study was the isolation of a probe set that would hybridise directly across species that diverged ~255 mya. More limited success in hybridising to *A. carolinensis* metaphases (2*n* = 36) (Fig. 1) revealed some broad similarities to the DCA established by the bioinformatic approach (see Supplementary Data 3).

### FISH analysis suggests avian and turtle chromosomes are precise counterparts

Cross-species hybridisation (zoo-FISH) of chicken bacterial artificial chromosome (BAC) probes previously designed to work in multiple avian species and located subtelomerically^5^, as well as chicken chromosome paints were successfully hybridised to the chromosomes of *A. carolinensis* (Carolina anole lizard), as well as two turtles *T. scripta* and *A. spinifera*. Results, for the most part, provide evidence that, for avian chromosomes 1–28 + Z (with rare exceptions, e.g. 16 for which we could not generate data), each chicken chromosome is syntenic to the turtle with the largest diploid number, *A. spinifera* (2*n* = 66). That is, we found little or no evidence of interchromosomal rearrangement, with the exception of rare events, e.g. fusions of chromosome 4 in chicken and chromosome 22 in turtles. For the macrochromosomes and some pools of microchromosomes, chromosome paints^18^ produced signals cross-species and, for the microchromosomes, selected BAC probes^5^ provided strong BAC signals (note, macrochromosomal BACs, despite working well on other avian species, did not produce many successful hybridisations on non-avian reptiles). Specifically, of the microchromosomal BACs, 29 of the original 36 (81%) worked successfully (chromosomes 10–15 and 17–28; 16 not included due to lack of sequence coverage and chromosomes smaller than 28 do not have sequences assigned to them) in both turtle species (all but chromosome 20 had at least one BAC signal) and 17 of 36 (47%) worked in Carolina anole lizard (chromosomes 14, 18, 20, 25, 27 and 28 were not represented). Of those that worked on all species, results revealed that the orthologues of chicken chromosomes 12 and 13 were fused and chromosome 26 attached to chromosome 4 in the red-eared slider and Carolina anole but represented as separate microchromosomes in the spiny soft-shelled turtle (*A. spinifera*). The chromosome 22 orthologue appeared as a separate chromosome in the Carolina anole, but as fused to the centre of a macrochromosome in the two turtle species. Eight avian microchromosome orthologues for chromosomes 10, 11, 15, 17, 19, 21, 23 and 24, appeared to be conserved as single microchromosomes in all three reptiles studied, and in red-eared slider (*T. scripta*), chromosomes 14, 18, 25 and 28 were also represented as single microchromosomes, with chromosomes 12, 13 and 26 also as single microchromosomes for spiny soft-shelled turtle. All macrochromosomal assignments confirmed those previously reported for the lizard and for the two turtles. In order to establish that there were no rearrangements between microchromosomes of birds and reptiles, working BACs as close to the telomere as possible were used, and all microchromosomal sizes were measured in comparison to known macrochromosomal size by ImageJ. We cannot preclude the possibility that some interchromosomal events were not detected by our approach; for instance, cryptic translocations could possibly give chromosome paint signals too weak to detect microscopically or be sub-telomeric to the BAC used. However the fact that no additional rearrangements other than those already known were detected between *A. spinifera* (2*n* = 66) and avian (2*n* = 80) karyotypes supports our central assertion of identity by descent, in place ~255 mya, with ~7 fissions (presumably at least some, but possibly all) occurring before the emergence of the dinosaurs.

### Sequence alignments, multispecies HSBs and EBRs

Pairwise bioinformatic alignments of the chicken, mallard, zebra finch, Carolina anole and grey short-tailed opossum genomes using the Evolution Highway chromosome browser allowed for the visualisation of multispecies HSBs (msHSBs) and their orientation in each genome as well as the identification of EBRs between these msHSBs. These alignments were screened for blocks shared in the five genomes compared, and a total of 397 msHSBs were found. These were distributed across 19 of the 28 sequenced chicken chromosomes available on Evolution Highway: i.e. chromosomes 1–9, 11–13, 15, 18, 20, 24, 26, 27 and Z. The 397 msHSBs were also dispersed on 19 duck chromosomes, 21 zebra finch chromosomes, 10 anole chromosomes, and 8 opossum chromosomes. If we compare the total size of 397 msHSBs relative to the chicken 28 chromosomes available on Evolution Highway, they represented 49% of the total genome length; if we compare these msHSBs to the size of the above 19 chicken chromosomes, they represented about 53% of their combined length.

Using the MGRA2 algorithm, we produced a series of CARs representing the most likely ancestral karyotype of the diapsid common ancestor (DCA). While we cannot be entirely sure that each CAR represents a whole chromosome as MGRA2 will inherently ‘break’ the chromosome if it cannot find synteny, a total of 19 diapsid ancestral CARs were found, probably representing fewer chromosomes. The number of msHSBs per CAR varied between 2 and 59. A total of 17 chicken chromosomes were aligned to these CARs (chromosomes 1–8, 11–13, 15, 18, 24, 26, 27 and Z) meaning that some microchromosomes were not represented.

### Chromosome inversions from DCA to chicken

**Fig. 2:**
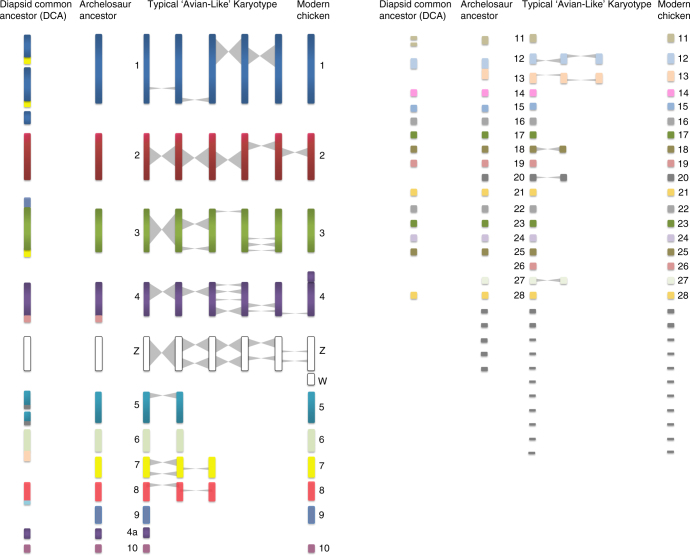
Overall inferred karyotypic changes from the diapsid common ancestor (DCA), through the archosauromorph ancestor, modern chicken (2*n* = 78) arising out of theropod dinosaurs, collating all available lines of evidence, bioinformatic and molecular cytogenetic. The left side shows the 19 CARs including the ancestral microchromosomes, which gives an impression of a higher diploid number greater than 46 (total chromosome number predicted) as we cannot necessarily determine the nature of all the fusions. For simplicity, the intrachromosomal rearrangements (inversions) are all depicted after the archelosaur ancestor, however, a small proportion may have occurred before. The colour scheme is randomly assigned with each chromosome for which we have data given a different colour. Chromosomes for which we do not have FISH data are depicted in grey (e.g. chromosomes 16, 20, 22 and the very smallest microchromosomes). Diapsid common ancestor (DCA): 255 mya; likely 2*n* = 36–46; 19 CARs identified (some CARs likely fused as single chromosomes, hence this diagram appears as apparently more chromosomes; some chromosomes not covered by sequence assembly; Chromosome 7 orthologue (yellow) attached to three chromosomes, chromosome 26 (dark pink) to the orthologue of chromosome 4). Archelosaur ancestor: <255 mya**;** likely ~2*n* = 66; most chromosomes syntenic to modern birds; chromosomes without direct evidence (including chromosome 16, 20, 22) depicted in grey. Typical ‘Avian-Like’ karyotype: likely ~2*n* = 80 (numbering according to chicken genome); ~7 fissions; some inversions may have occurred before the pattern was established interchromosomally, but intrachromosomal changes shown separately for clarity. Modern chicken: 2*n* = 78; known fusion of chromosome 4 shown (not present in most birds); sex chromosome evolution post Palaeognathae–Neognathae divergence

Reconstructed CARs derived from MGRA2 were subsequently mapped to the extant genomes. The rearrangements between the DCA and chicken were then modelled using maximum parsimony. A total of 49 inversions were identified between the DCA and the chicken genome along with 10 interchromosomal changes (see Supplementary Note 3). Of the interchromosomal rearrangements found, a translocation was identified between orthologues of chicken chromosomes 5 and 20, consistent with the FISH results (Fig. 2, Supplementary Fig. 1). Comparison of the data generated for the DCA and a putative archelosaur common ancestor revealed that the majority of interchromosomal rearrangements occurred to form this basic structure with most intrachromosomal rearrangements (inversions) after (for simplicity, on Fig. 2, all intrachromosomal changes are shown after formation of the basic (archosauromorph common ancestor) pattern) although we cannot rule out the possibility that some occurred before. Between the DCA and the archosauromorph common ancestor, a fusion most likely occurred to form chromosome 1 and translocations/fissions occurred between avian ancestral CARs that became chromosome 7). Most rearrangements between these two ancestors were nonetheless intrachromosomal with a total of 49 inversions that appear to have occurred between the DCA and the extant chicken genome.

### Enrichment of gene ontology terms in msHSBs

Within the 397 multispecies HSBs (msHSBs), significant enrichments were observed for gene ontology (GO) terms relevant to amino acid transmembrane transport (symport; Supplementary Data 4) and signalling (group enrichment scores, 2.44–2.50 as produced using DAVID Functional Annotation Clustering tool; single GO terms as produced using DAVID Functional Annotation Chart, *p* < 0.05, false-discovery rate (FDR)  < 5%). Other msHSB-specific GO term enrichments were related to synapse/neurotransmitter transport, nucleoside metabolism and use, cell morphogenesis and cytoskeleton and sensory organ development (group enrichment scores, 1.72–3.02––Supplementary Data 4). For the functional analysis of EBRs, we produced a set of 234 EBRs that were intrinsic to the archosaur common ancestor. Within these, we identified significant enrichments in genes and single GO terms relevant to chromatin modification and chromosome organisation, as well as proteasome/signalosome structure (*p* < 0.01, FDR < 5%, Supplementary Data 5). In particular, the first annotation cluster (enrichment score, 4.30) consisted of six genes related to proteasome/signalosome structure within EBRs located on six chicken chromosomes. The second annotation cluster (enrichment score, 1.64) included 15 genes relevant to chromatin modification within 12 EBRs on seven chicken chromosomes, these genes and their functions are listed in Supplementary Data 5.

## Discussion

**Fig. 3:**
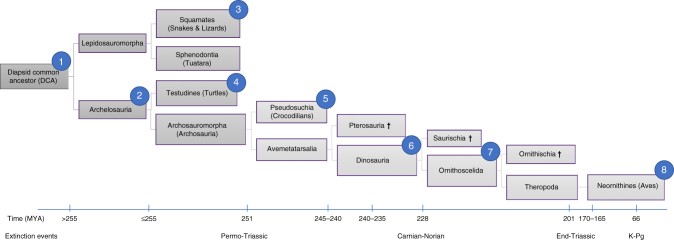
Representative phylogenetic tree illustrating the lineage investigated in this analysis. For ease of reading, the timelines are not to scale. The major extinction events and the principal findings of this study are highlighted in the context of this phylogeny as follows. (1) DCA karyotype reconstructed by MGRA2 analysis. (2) Cytogenetic analysis revealed the basic 'avian' pattern 2*n* = 66 mostly in place before archelosauria divergence. (3) One lizard (*A. caroliensis*) studied genomically and cytogenetically. (4) Two turtles (*T. scripta* and *A. spinifera*) studied cytogenetically. (5) Crocodilian genomes not considered suitable for analysis because of fragmented genome assemblies and fused chromosomes. (6) Early emerging dinosaurs and pterosaurs probably had at least 2*n* = 66 and up to 2*n* = 80 with typical avian pattern. (7) Theropod dinosaurs and possibly other groups probably had close to 2*n* = 80 with typical avian pattern. (8) Three avian species 2*n* = 78–80 studied cytogentically and using chromosomal-level genome assemblies. † extinct lineage

Combining all lines of available evidence (bioinformatic and molecular cytogenetic from this study along with previous findings), a picture emerges of an inferred DCA karyotype with a chromosome number of 2*n* = 36–46, consistent with that previously proposed^19^ and with most other non-avian, non-testudinate reptiles. Roughly half would have been macro- and half microchromosomes^6,19^. Alföldi et al.^6^ found direct synteny at the sequence level between the microchromosomes of *A. carolinensis* and *G. gallus*, with all but one lizard microchromosome corresponding to a single chicken microchromosome. Given that the *A. carolinensis* genome has 12 microchromosomal pairs (that are mostly syntenic to chicken chromosomes^6^) compared to the 28–30 seen in most birds, the most likely explanation is that these were present in the DCA, the remainder evolving thereafter by fission^6^ to at least 2*n* = 66. Our current data suggest that interchromosomal rearrangement largely ceased thereafter, with the exception of ~7 fissions that explain the difference between the pattern in *A. spinifera* (2*n* = 66) and most modern birds (2*n*~ = 80). Indeed, even the karyotype of *T. scripta* (2*n* = 50) has broad similarities to the avian pattern, but with more microchromosomal homeologues attached to larger chromosomes. This would either indicate that *T. scripta* has a karyotype that represents an earlier stage of differentiation to the “bird-like” turtle pattern, or that it subsequently underwent a series of fusions (such as in the crocodilians); the former being the most likely since it requires fewer events. The considerable range of diploid numbers in turtles (2*n* = 26 to 2*n* = 68), with most being more 'avian-like' than their other reptilian counterparts, suggests that further study of this group will provide greater insight into the sequence of events that led to the establishment of a highly successful mode of genome organisation. We cannot preclude the possibility that some interchromosomal rearrangements were beyond the sensitivity of our detection, e.g. the weak chromosome painting signals may not have detected subtle changes, or cryptic chromosome translocations may have occurred that were telomeric to our fluorescent probes. This does not, however, detract from our assertions that a broad overall pattern of genome organisation was in place at least 255 mya in the archelosaur ancestor and changed little in the majority of living species. Moreover, our findings are consistent with previous studies using chicken macrochromosome paints on Chinese soft-shelled turtle (*Pelodiscus sinensis*) chromosomes (2*n* = 66)^20^, *T. scripta*^21^ and painted turtle (*Chrysemys picta*) chromosomes (both 2*n* = 50)^22^. Thus, similar studies on more turtles may reveal species with greater diploid numbers and a pattern resembling that of birds even more (Figs. 2 and 3).

Determining the precise timing of events that led from the archelosaur ancestor (2*n* > 66) to modern birds (2*n*~ = 80) is beyond the resolution of this study (Fig. 3). A similar rate of fission continuing beyond 255 mya, would have established a near-complete neornithine-like karyotype by ~240 mya, roughly coinciding with the emergence of the earliest dinosaurs and pterosaurs^23^. Equally, a dramatic slowdown or halt in fission events at ~255 mya would suggest that the forebears of the earliest dinosaurs and pterosaurs had a pattern more similar to that of *A. spinifera*. In both scenarios (or an intermediate), however, a pattern very similar to that of most birds would have been present. Burt^24^ proposed that most avian microchromosomes were present in the avian common ancestor >80 mya^25^, arguing that it probably had the small genome size characteristic of birds and a karyotype of around 2*n* = 60. Our results, however, suggest a much earlier emergence of the typical avian pattern with genome size reduction occurring later in archosaur or theropod evolution^24^. Indeed Uno et al.^26^ proposed that the archosauromorph ancestor may have had microchromosomes similar to the turtle but did not specify their nature.

Our results suggest that, aside from ~7 fissions, the primary mechanism for chromosomal rearrangement in the avian stem lineage after 255 mya was intrachromosomal (e.g. inversions). Reconstructed CARs, when compared to extant genomes, resulted in the identification of rearrangements between the DCA and chicken (*G. gallus)* genomes, modelling 49 inversion events. This, however, is almost certainly an underestimate due to the paucity of sequence coverage in some areas, including the smallest avian microchromosomes. Rates of change are not easy to determine, but there is some evidence of intrachromosomal change speeding up in modern birds, even in the chicken, which is thought to be very similar chromosomally to the avian common ancestor^27^. Increased intrachromosomal change has been reported in specific groups, with several studies suggesting that the greatest rates would be found in passerines (compared to other birds)^1,4,28^, the group containing most avian species. It is perhaps reasonable to speculate therefore that bursts of speciation may have also been accompanied by increased rates of intrachromosomal rearrangement in other non-avian dinosaur lineages.

Within the multispecies HSBs, we identified significant enrichments for GO terms relevant to transmembrane transport (symport) and signalling, synapse/neurotransmitter transport, nucleoside metabolism and use, cell morphogenesis and cytoskeleton and sensory organ development (Supplementary Data 4). HSBs are often enriched for GO terms related to phenotypic features that remain constant^29^ and the results presented here are consistent with this hypothesis. Sankoff^30^ however stated that EBRs are where the ‘action’ in genome evolution lies and, previously, we found that GO terms in avian EBRs associated with specific adaptive features, e.g. enrichment for forebrain development in the budgerigar EBRs (consistent with vocal-learning)^4^. In the current study, we identified significant enrichments in genes and single GO terms relevant to chromatin modification, chromosome organisation and proteasome/signalosome structure in EBRs (Supplementary Data 5). This illustrates some parallels to recent findings in rodents where chromosomal changes were associated with open chromatin^27^. Most of the 15 genes found in this GO term were related to control of gene expression. Transcription factors modify chromatin by making it accessible during transcription and this is noteworthy because EBRs rearrange transcription factor genes. This might affect expression of other genes of the same pathway. Interestingly, two of these genes showed a different expression pattern between birds and mammals, HDAC8 involved in early embryo development^31^ and PRMT8 expressed in brain^32^. These results suggest a correlative link between chromosomal and morphological changes among species, mediated by rearranging genes controlling the expression of developmental pathways.

Estimates of non-avian dinosaur genome size, based on osteocyte sizes inferred from bone histology, identified a distinction between small, characteristically avian, genomes in theropods and sauropodomorphs vs. much larger ornithischian genomes^33^. These results were interpreted as supporting the hypothesis that small genome size and low repeat content was a genomic exaptation that preceded and facilitated the endothermic metabolic demands of birds, e.g. for flight^34^. It was further hypothesised that the avian karyotype evolved in response to a reduction in genome size in birds^34^. This theory was subsequently challenged by a study that suggested that a decline in overall genome size occurred in non-volant dinosaurs^33^. Here, we propose further that an avian-like karyotype not only predated the origin of flight but evolved well before, and independent of any, purported genome size reduction. Nonetheless, we note that there may be an association between genomes with fewer chromosomes (and no microchromosomes) and larger genome sizes around 2.5–3 Gb, as in mammals^35^ and crocodilians^15^. More repetitive elements could provide substrates for interchromosomal rearrangement, which is commonplace in mammals but rare in birds, and it has been suggested that an avian karyotype provides fewer opportunities for interchromosomal rearrangement due to the existence of fewer recombination hotspots (despite an overall higher recombination rate)^36,37^, repeat structures^12,38,39^ and endogenous retroviruses^2,4,40^. Therefore, although flight evolution might be correlated with smaller genome size (consider pterosaurs vs. other avemetatarsalians^41^; bats vs. other mammals^34^ and strong vs. weak flying/ flightless birds^42^), other mechanisms are clearly involved. Specifically, the formation of an avian-like karyotype long before the evolution of flight suggests neither a causative, nor a correlative link between the two.

Stasis of this karyotypic structure for >255 million years nonetheless suggests a highly successful mode of genome organisation that might have provided a blueprint for evolutionary success. The reasons for its persistence are speculative but might be due to its ability, facilitated by many chromosomes, including microchromosomes with high recombination rates, to generate variation, the substrate of natural selection. A larger number of small chromosomes inherently generate variation through increased genetic recombination and increased random chromosome segregation. Burt^24^ suggested that a higher recombination rate has also contributed to the unique genomic features seen in microchromosomes such as high GC-content, low repeat content and high gene-density, which subsequently led to its maintenance. Variation, in turn, facilitates rapid adaptation and may therefore have contributed to wide phenotypic variation, in extant animals represented by >10,000 species of birds (2*n*~ = 80), >300 species of turtles (2*n* < 68) and, quite possibly, a large number of non-avian dinosaurs also. Of course, a karyotype with many, tiny chromosomes is not the only means by which variation can be generated (genic, epigenetic and interchromosomal variation all are mechanisms reported in other groups): indeed, amphibians display enormous phenotypic variation but possess relatively few chromosomes. Nonetheless, the above may explain the apparent paradox of a group with very little interchromosomal change, but incredible phenotypic diversity.

In conclusion, any 'dinosaur genomics' effort of this type is limited to reconstructing common ancestors (e.g. of birds and crocodilians), along with other nodes that have extant descendants (e.g. Archelosauria, Diapsida etc.) and inferring the most parsimonious set of events that led to extant animals. With this in mind, few studies have attempted to infer the nature of the gross structural genomic changes that occurred from the DCA, to the archelosaur ancestor, to birds. Given our data, it is perhaps not an unreasonable speculation that, if we had the opportunity to make metaphase chromosomes from tissue of non-avian theropods, both karyotypic and molecular cytogenetic analysis (genome size aside) would reveal little difference from a modern chicken, duck or ostrich (or at least a spiny soft-shelled turtle), i.e. 2*n* = 66–80 in the majority of species. Of course, we cannot preclude the possibility that certain groups of non-avian dinosaurs underwent significant interchromosomal change, as these are known to occur among extant avian dinosaurs (kingfishers^43^ (fissions), parrots^44^ and falcons^5^ (fusions) are modern examples). Rather than being simply interesting descriptions of inferred karyotypes, therefore, we propose that the overall genome organisation and evolution of dinosaur chromosomes (inclusive of the avian radiation) might have been a major contributing factor to their morphological disparity, physiology, high rates of morphological change^45^ and ultimate survival. In other words, we have an apparent paradox of a highly stable karyotype that is rarely changing, nonetheless contributing to great morphological diversity. We already believed this to be the case for the great phenotypic diversity we see in birds; the current results however suggest that the karyotype may have contributed to species diversity in non-avian dinosaurs also. Moreover, the evidence that the karyotype had deeper origins than previously appreciated is congruent with other recent discoveries about dinosaur morphology, demonstrating that features previously thought to be characteristic of crown-group birds only (e.g. feathers and pneumatised skeletons) arose first among more ancient dinosaur or archosaurian ancestors^23,46^. Dinosaurs have pervaded popular culture and the creative arts, perpetuated, in part, through film, television, press and literature. Their dominance for many millions of years, their radiations following two mass extinction events and, despite being almost wiped out by a third (the K–Pg meteor impact), their persistence as a highly diverse and speciose clade (extant birds)^47^ has fascinated scientists since the very earliest discoveries. Of course, many of the evolutionary changes were in response to a rapidly changing environment. Whether a disproportionate advantage was offered by having an ‘avian-like’ karyotype will be the subject of future studies and speculation.

## Methods

### Cell culture and chromosome preparation

Chromosome preparations were established from fibroblast cell lines of the Caroline anole (*A. carolinensis*) (2*n* = 36), red-eared slider (*T. scripta*) (2*n* = 50) and spiny soft-shelled turtle (*A. spinifera)* (2*n* = 66). Cells were cultured at 30 °C and 5% CO_2_ in Alpha MEM (Fisher), supplemented with 10% fetal bovine serum (Gibco) and 1% Pen-Strep-l-gutamine (Sigma). Chromosome suspension preparation followed standard protocols, briefly, mitostatic treatment with colcemid at a final concentration of 5.0 μg/ml for 1 h at 40 °C was followed by hypotonic treatment with 75 mM KCl for 15 min at 37 °C and fixation with 3:1 methanol:acetic acid.

### Selection of BACs

Chicken and zebra finch BACs were chosen for interspecies FISH experiments according to a range of criteria, including the proportion of conserved elements shared across multiple avian species. Due to the high degree of apparent genome conservation observed between avian and reptilian species, this set of BACs was applied to chromosome suspensions of the birds in this study and from *A. carolinensis*, *T. scripta* and *A. spinifera*.

### Preparation of BAC clones for FISH

BAC clone DNA was isolated using the Qiagen Miniprep Kit (Qiagen) prior to amplification and direct labelling by nick translation. Probes were labelled with Texas Red-12-dUTP (Invitrogen) and FITC-Fluorescein-12-UTP (Roche) prior to purification using the Qiagen Nucleotide Removal Kit (Qiagen).

### Fluorescence in situ hybridisation

Metaphase preparations were fixed to slides and dehydrated through an ethanol series (2 min each in 2×SSC, 70%, 85% and 100% ethanol at room temperature). Probes were diluted in a formamide buffer (Cytocell) with Chicken Hybloc (Insight Biotech) and applied to the metaphase preparations on a 37 °C hotplate before sealing with rubber cement. Probe and target DNA were simultaneously denatured on a 75 °C hotplate prior to hybridisation in a humidified chamber at 37 °C for 72 h. Slides were washed post hybridisation for 30 s in 2 × SSC/0.05% Tween 20 at room temperature, and then counterstained using VECTASHIELD anti-fade medium with DAPI (Vector Labs). Images were captured using an Olympus BX61 epifluorescence microscope with cooled CCD camera and SmartCapture (Digital Scientific UK) system. Fissions and/or translocations were detected if cross-species signals appeared on two different chromosomes on the species of interest, and fusions were identified where the signals appeared on a noticeable larger chromosome than the (usually chicken) chromosome from which it was derived. This was achieved by visual inspection aided by ImageJ analysis for the smaller chromosome.

### Reconstruction of the DCA karyotype

In order to reconstruct the hypothetical DCA, we selected the following sequenced extant amniote genomes: four diapsids (three birds, one lizard) and one basal mammalian representative as an outgroup (grey short-tailed opossum, *M. domestica*; assembly MonDom5). The three avian genomes, chicken (*G. gallus*; assembly galGal4), mallard (*A. platyrhynchos*; assembly BGI_duck_1.0; chromosome-level assembly, Faraut et al., personal communication) and zebra finch (*T. guttata*; assembly taeGut1), were also used to reconstruct the avian ancestor, in this case using the Carolina anole genome (*A. carolinensis*; assembly anoCar2) as the outgroup. In order to compare reptilian and avian genomes cytogenetically, we selected chicken BAC probes designed to work in FISH experiments on all avian and reptilian chromosomes^5^. By combining novel bioinformatic and FISH data produced for the current study with that of previous studies, we established, by inference, the most parsimonious explanation of the available data regarding the nature of the dinosaurian genome. Initial experiments designed to establish whether the sequenced crocodile genomes^48^ were suitable outgroup species met with only limited success, given the fragmented nature of these assemblies. This, and the fact that the microchromosomes are fused in extant crocodilians (2*n* = 30) meant that this genome assembly was not a suitable reference for our work; there was also no sufficiently well-assembled chromosome-level turtle genome available for analysis at the time of writing.

### Alignment of multiple genomes and identification of HSBs and EBRs

Results from this study were generated from the alignment of the three best avian genomes assembled at a chromosomal level (chicken, mallard and zebra finch) along with the best-assembled reptile genome available assembled to a partial chromosomal level (Carolina anole) and one mammalian outgroup, grey short-tailed opossum (all genomes were aligned against chicken). The whole-genome sequences of the species of interest were aligned using LastZ and visualised using the interactive genome browser Evolution Highway^4, 49^. Pairwise blocks of synteny were identified relative to chromosomes of the chicken, which served as a reference genome (galGal4). Genome alignments for the five species as inferred from sequence orthology maps were mapped against chicken chromosomes. The start and end coordinates of the contiguously aligned orthologous regions observed in all the species compared were used to define msHSBs at the 300-kb resolution. These msHSBs were assigned to and subsequently sorted in individual chromosomes in each species according to their location, orientation and sequential order.

### Arrangement of ancestral diapsid and avian karyotypes

To reconstruct a putative ancestral DCA karyotype, the Multiple Genomes Rearrangements and Ancestors tool version 2 (MGRA2^50^; http://mgra.bioinf.spbau.ru/), was used as follows: based on pairwise alignments for mallard, zebra finch, Carolina anole, and grey short-tailed opossum visualised relative to the chicken, a set of respective msHSBs was generated as referred to above. In this case, the orthology map of the opossum was used as an input for the MGRA programme and included in the analysis as an outgroup. The five species-specific msHSB sets served as MGRA2 inputs for individual genomes which then produced a series of CARs representing the most likely ancestral configuration for the species identified in both hypothetical diapsid and avian ancestors.

### Genome rearrangement analysis

To reconstruct the chromosomal changes that occurred between the groups, we used the MGR and Genome Rearrangements In Man and Mouse (GRIMM tools^51^; http://grimm.ucsd.edu/). MGRA2 outputs served as MGR/GRIMM inputs to trace the most parsimonious scenarios for evolutionary changes in two scenarios: first, the intrachromosomal and interchromosomal rearrangements that might have occurred from the hypothetical diapsid ancestor to the avian one and second, those rearrangements that may have occurred between the avian ancestor and the extant species.

### Identification of gene ontology enrichment terms in HSBs and EBRs

Gene lists for msHSBs and archosaur EBRs were extracted from the Ensembl BioMart data system using chicken as the reference. With the chromosome-level assemblies available on Evolution Highway, definitions of EBRs are only possible for the archosaur ancestor. Since human genes are best annotated, the gene lists derived from chicken were matched to orthologous human genes and filtered for homology type and orthology confidence, leaving only those genes that were one-to-one orthologues. Background gene lists were also generated using all chicken–human orthologues with the maximum orthology confidence. The first two background gene lists tested covered all assembled chicken chromosomes and the second two lists only included results for 19 of the chicken chromosomes where the msHSBs and EBRs were found. In addition, in order to test whether genes with low gene identity matches affected the GO analysis, thresholds of 70, 60 and 50% homology at nucleotide level for the orthologue gene lists were set and the resulting GO outputs were compared. Based on these tests, the 70% gene identity threshold was selected for generating the msHSB/EBR gene lists, and the 19-chromosome list with all orthologous genes was used for the background GO analysis list. Gene lists were used as inputs for the web-based functional annotation tool DAVID^52^ using human Ensembl Gene ID as the list identifier and subsequently analysed using the Functional Annotation Clustering tool. Cluster data from each gene list output were downloaded into Microsoft Excel and filtered using an enrichment score of ≥ 1.3 and a *p*-value < 0.05 to edit the list for clusters considered to be significant. In addition, Functional Annotation Chart reports containing single GO terms and their associated genes were generated using the same gene lists. The latter information was also taken into account to identify significant GO terms for the tested gene lists, especially in situations when the Functional Annotation Clustering analysis did not result in any significant gene–GO term enrichment groups. In order to correct for multiple sampling error, a FDR threshold of 5% was used. Finally, individual lists of genes that fit the GO criteria were manually curated and their function established, initially from Ensembl and thereafter from the original publications that described their isolation and analysis.

### Data availability

The authors declare that the data supporting the findings of this study are available within the paper (and its supplementary information files).

## Electronic supplementary material

Supplementary Information

Description of Additional Supplementary Files

Supplementary Data 1

Supplementary Data 2

Supplementary Data 3

Supplementary Data 4

Supplementary Data 5
