# Supplementary material for: Reconstruction of the diapsid ancestral genome permits chromosome evolution tracing in avian and non-avian dinosaurs
Source: Nat Commun. 2018 May 21;9:1883. doi: 10.1038/s41467-018-04267-9 (PMC5962605; doi:10.1038/s41467-018-04267-9)
Supplement: Supplementary file 1 — Supplementary Information [file 41467_2018_4267_MOESM1_ESM.pdf]

Supplementary Figure 1. Outputs of the MGRA data expressed as chromosome rearrangements. All arrows start pointing from left to right in the DCA karyotype. The numbers refer to those assigned to the HSBs with reference to the chicken genome (Supplementary data 2). In the chicken chromosomes therefore, the numbers are sequential. For instance, in the first panel (DCA1 ->GGA2; diapsid common ancestor evolving into *Gallus gallus* chromosome 2) the numbers are continuous from 97 to 148.

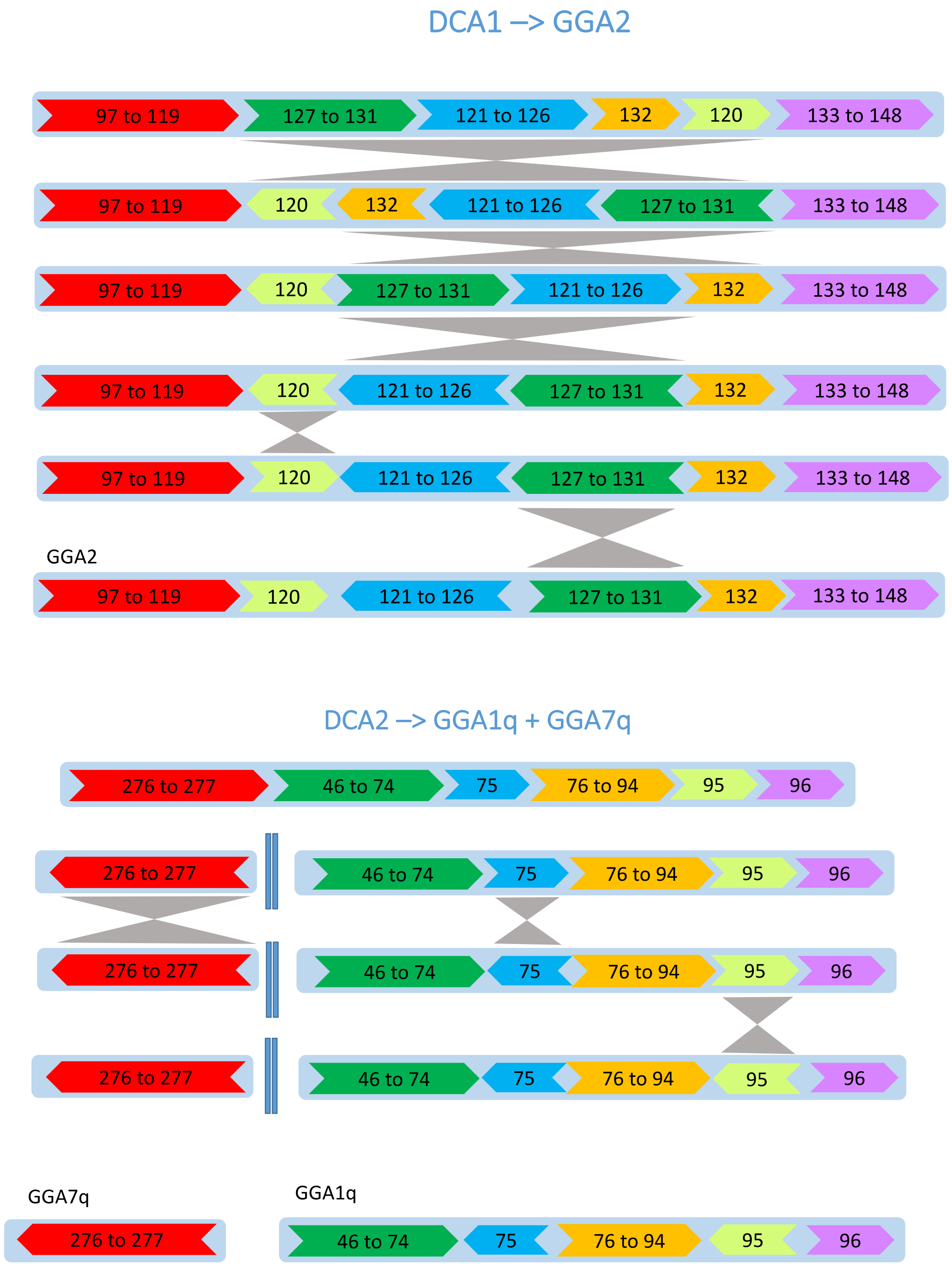

DCA3 –> GGA3p, GGA7p and GGA9

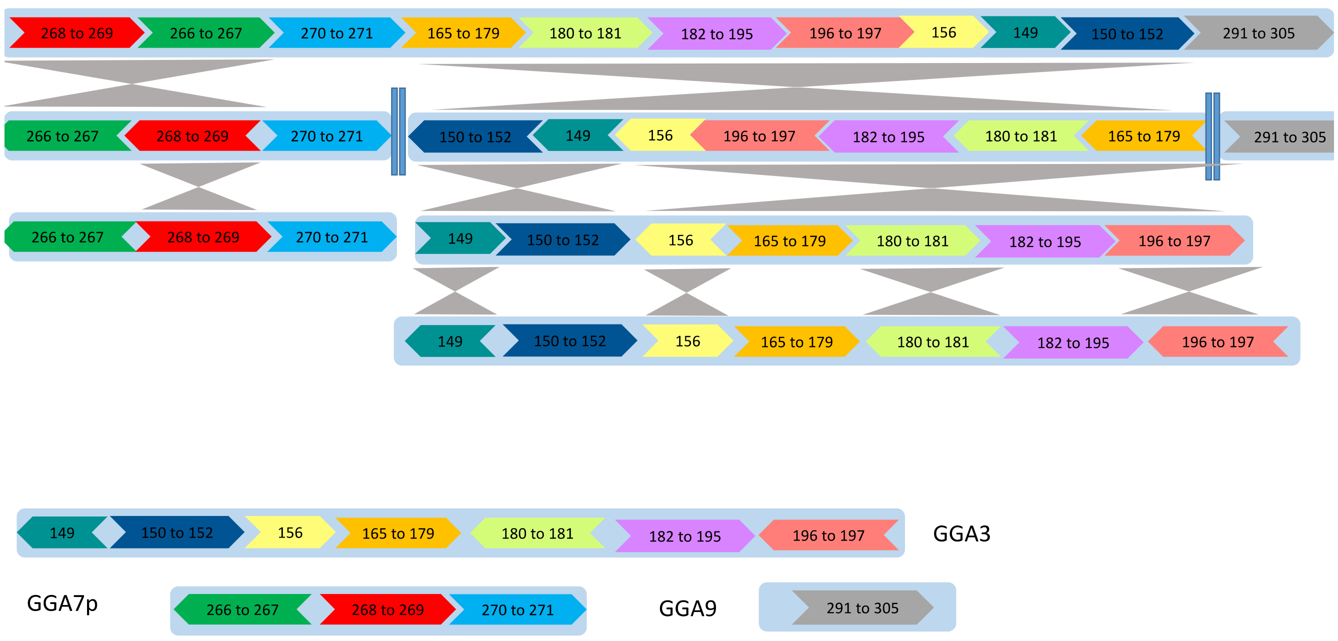

DCA4 –> GGA4

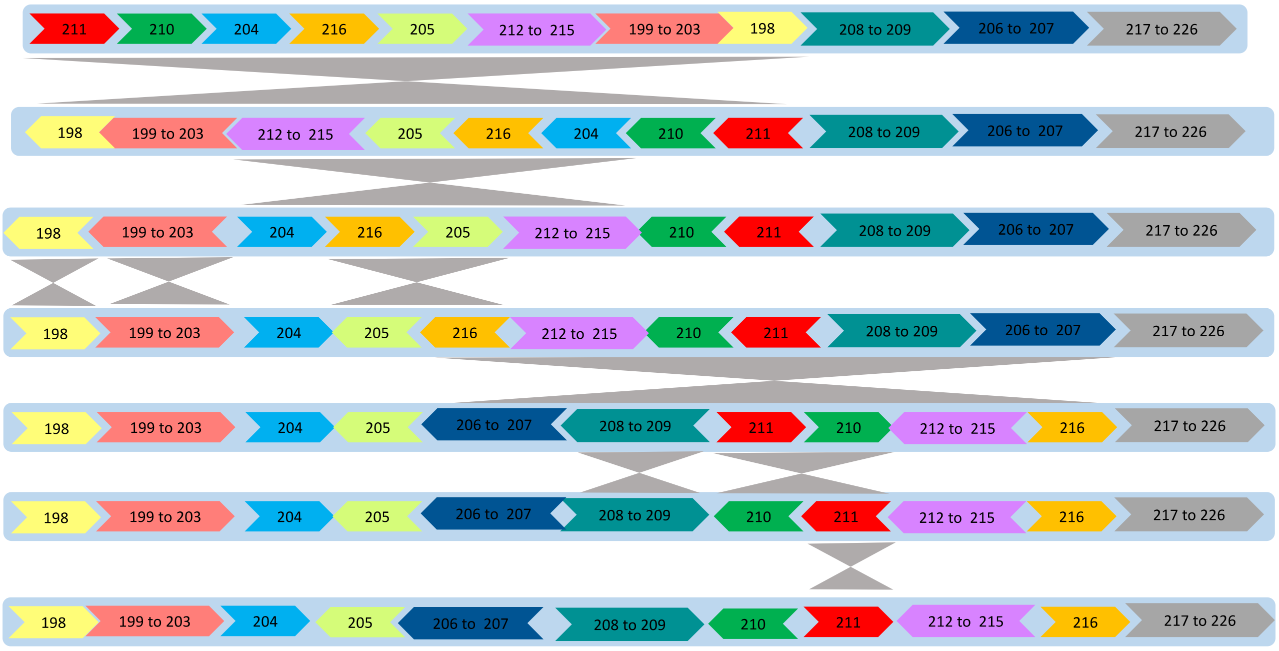

DCA5 –> GGA7mid and GGA1p

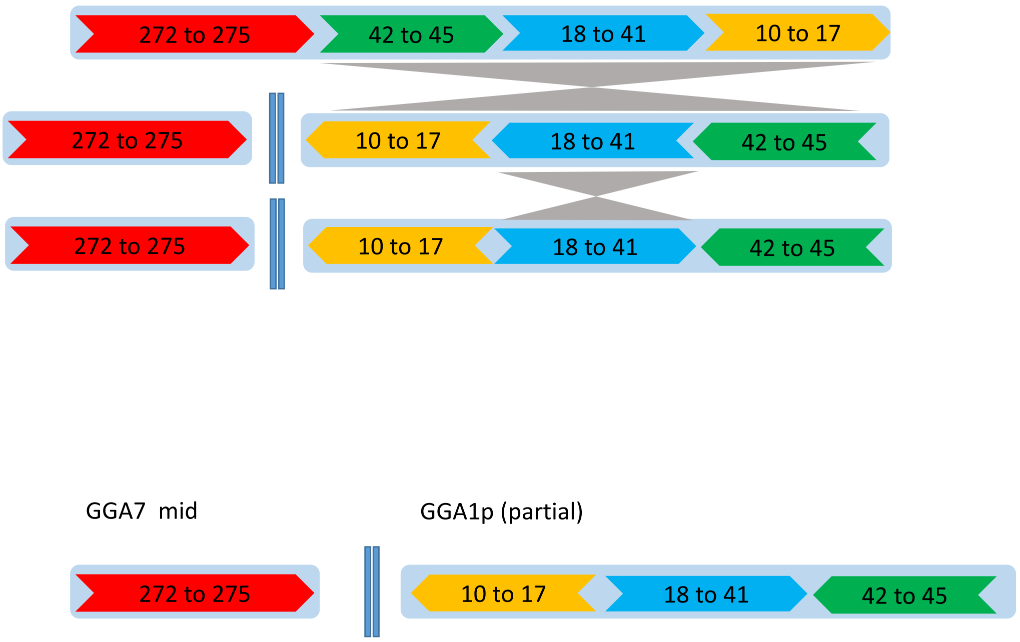

DCA6 and DCA7 –> GGA5 and GGA20

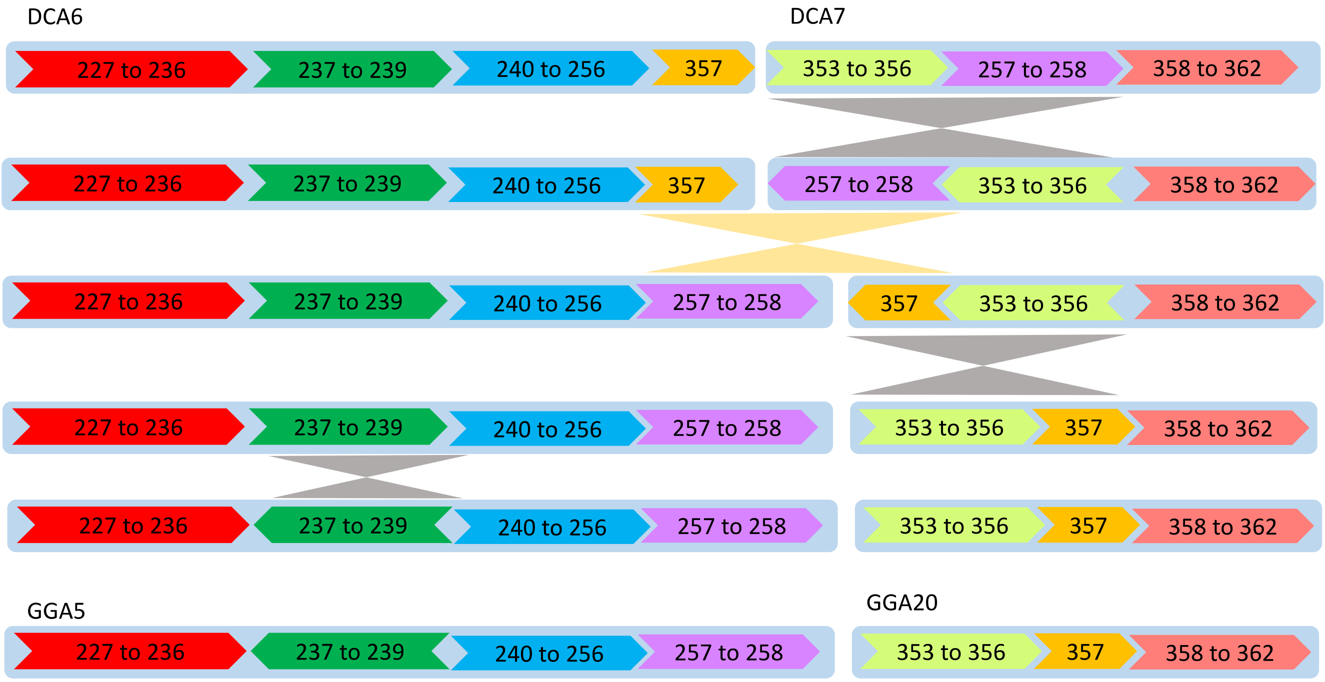

DCA8 –> GGA6 and GGA13

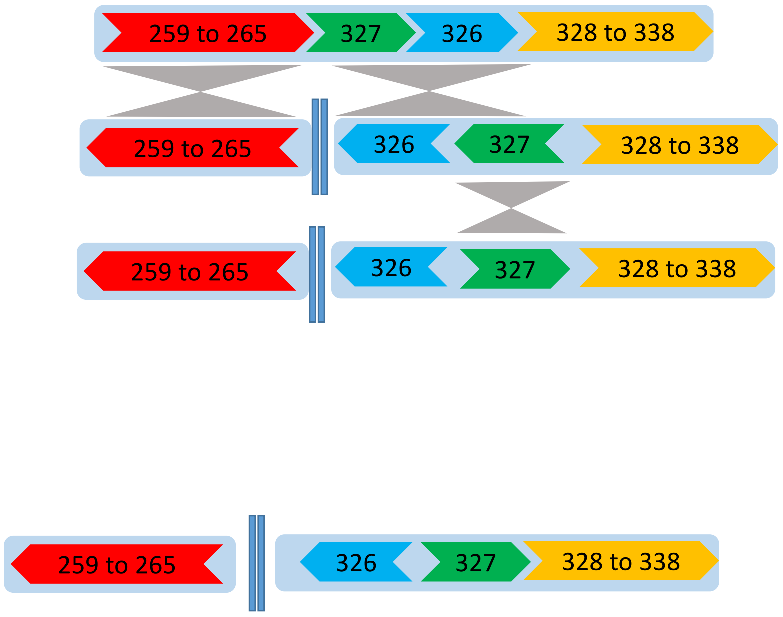

DCA9 –> GGA8 and GGA27

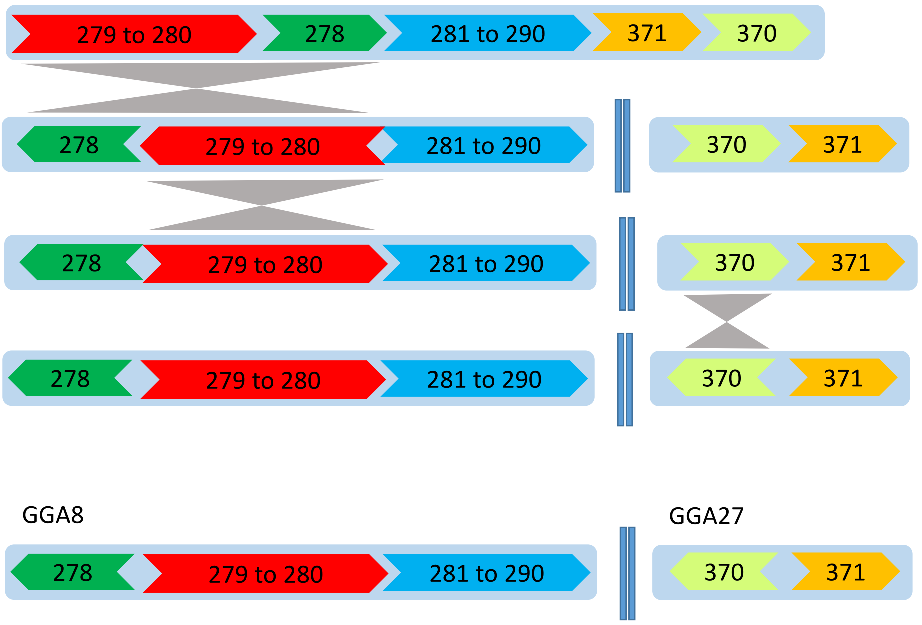

DCA10 –> GGA1p (partial)

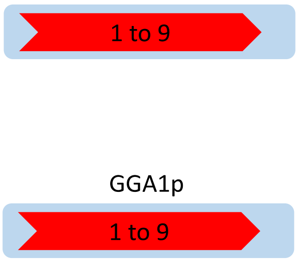

DCA11 –> GGA12

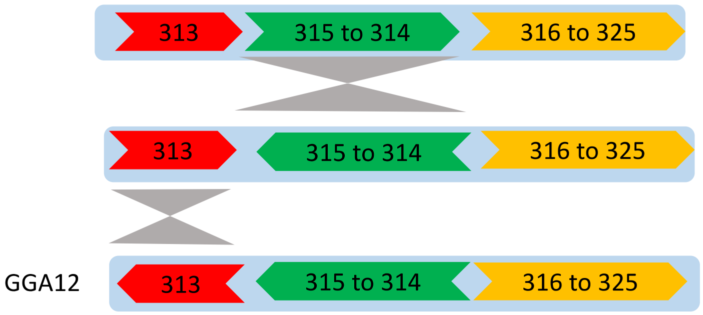

DCA12 –> GGA3p

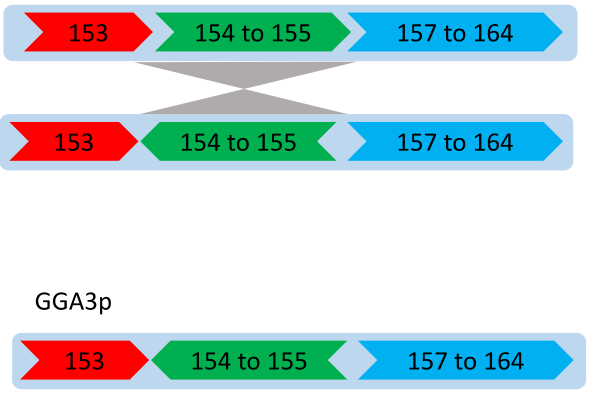

DCA13 –> GGA 18

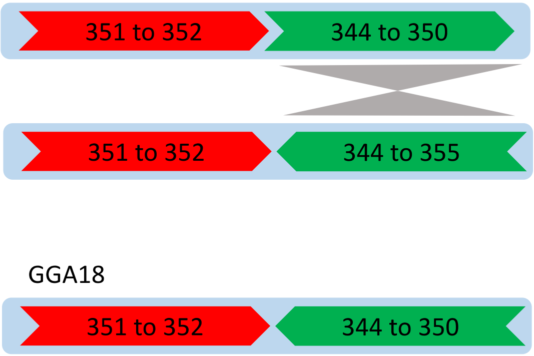

DCA14 –> GGA24

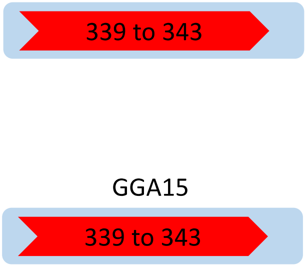

DCA15 and DCA16 –> GGA 11

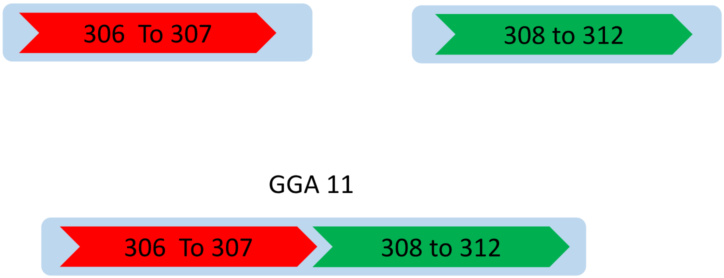

DCA17 – GGA24

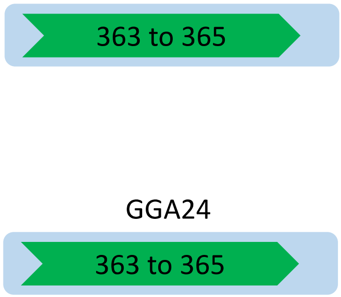

DCA18 – GGA26

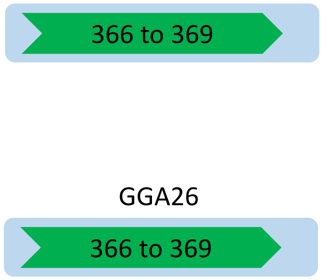

DCA19 –> GGAZ

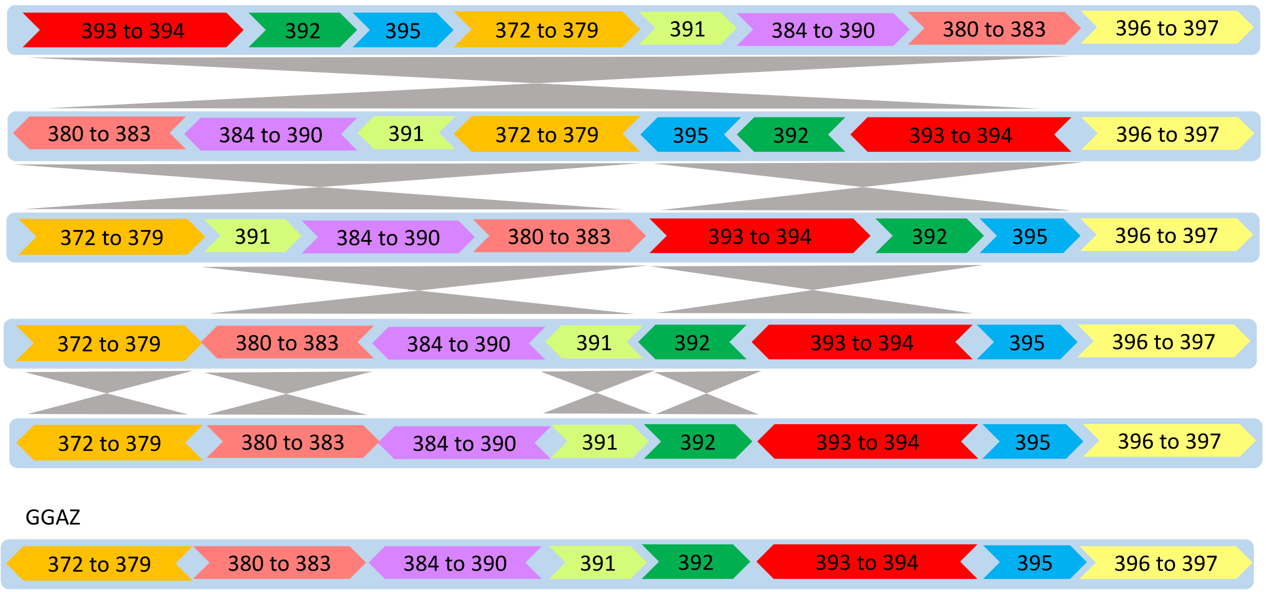

Reconstruction of the diapsid ancestral genome permits chromosome evolution tracing in avian and non-avian dinosaurs

O'Connor et al.

Supplementary Information

Supplementary Note 1: Key extinction events and divergence dates

Studies designed to date the divergence times between the various taxonomic groups that ultimately led to birds, from among the theropod dinosaurs, vary depending on the method employed. A reasonable summation of the most recent lines of evidence, however, suggests that the clade Archelosauria diverged from Lepidosauromorpha (snakes, lizards and their extinct relatives) >255 mya (million years ago). Archelosauria is a clade proposed within the last four years to include extant testudines (turtles), archosaurs (birds and crocodilians) and their fossil relatives, so is composed of the total groups Testudinata + Archosauromorpha. Archosauromorpha includes archosaurs (dinosaurs, including birds, pterosaurs, crocodilians, and all descendants of their common ancestors) and stem-archosaurs; Testudinata includes turtles and stem-turtles. The fossil record provides a similar soft minimum date for the Testudinata/Archosauromorpha divergence, but as this split is nested within Diapsida it must have occurred more recently in time, i.e. ≤255mya[1]. The Permo-Triassic mass extinction event (PTME) ~251 mya eliminated 80–90% of species[2] and preceded a period of extraordinary ecological change emergence of the two main clades within Archosauria, Pseudosuchia (crocodiles and their extinct relatives) and Avemetatarsalia (Dinosauromorpha, Pterosauria, and Aphanosauria) ~240–245mya[3]. Dinosaurs and pterosaurs characteristically evolved long hind limbs held erect beneath the body, diverging around this time from the Pseudosuchia (crocodiles and relatives). Dinosaurs had a relatively low species diversity and abundance until 220mya but, by the Middle Jurassic, their numbers, diversity, geographic spread, and body size increased significantly[4]. For 135 million years, they were the dominant vertebrate group, surviving various extinction events including the Carnian–Norian extinction event (CNEE) 228mya[5] and end-Triassic mass extinction event (ETME) 201mya, which devastated pseudosuchian diversity. Non-avian dinosaurs were wiped out by the Cretaceous-Paleogene (K-Pg) mass extinction event 66mya, but the neornithine birds radiated spectacularly in the wake of this extinction to achieve their current high levels of diversity[6]. Avian dinosaurs first appeared in the Late Jurassic (around 150mya), but phylogenetic evidence suggests that they might have diverged from other non-avian theropods a little earlier in time (by the early Late or Middle Jurassic, although fossils are currently lacking).

Among extant birds, Passeriformes constitute over 50% of species, with the Galliformes (particularly chicken) the best described cytogenetically and genomically. See Figure 3 for a diagrammatic explanation of this in the light of our data.

Supplementary Note 2: Genome organization and sequencing in archosaurs

Most reptiles (including birds) display a pattern of genome organization/structure (karyotype) that includes a small number of macrochromosomes (up to 10 pairs) and varying numbers of smaller, morphologically indistinguishable microchromosomes[7]. The exception is the crocodilians, which have no microchromosomes and have an average diploid number of $2n$=30[8]; comparison with other groups suggest these arose as a result of wholesale fusion. The only extant dinosaurs (Neornithes – modern birds) mostly have a very distinctive 'typically avian karyotype' of ~$2n$=80 containing ~10 macrochromosome and ~30 microchromosome pairs, more than any other terrestrial vertebrates. The microchromosomes include several that are physically smaller than in other reptiles, and many (chromosomes 29–38) remain to be sequenced or identified despite a few individual sequences that have been assigned putatively. Two-thirds of living birds have this basic pattern of genome organisation; another 25% of species are very similar in terms of overall numbers ($2n$=66-74); and there are several rare exceptions (e.g., Falconiformes and Psittaciformes) that have fewer chromosomes as a result of fusion. Cross-species chromosome painting (zoo-FISH) indicates that interchromosomal rearrangement is relatively rare, and most chromosomes are precise counterparts of one another from species-to-species in birds[7] and reports of chromosomes chromosomes 1–5 in Chinese soft-shell turtle (*Pelodiscus sinensis*) also apparently syntenic to chicken (*Gallus gallus*)[9]. To date, only chicken has had all its chromosomes characterised cytogenetically.

The first avian genome sequence (*G.gallus*) was published in 2004[10], with zebra finch[11] and turkey[12] both following in 2010, and duck in 2012[13]. In 2014, the publication of numerous avian genome sequences by the Avian Phylogenomics Group, took the total number of sequenced avian genomes to around 60[14]. These newly published genome sequences, along with previously published sequences, represent all 32 neognath orders and two of the paleognath orders giving nearly entire coverage (92%) of the major extant avian orders[14]. Concurrently, Green et al.[15] attempted to reconstruct an ancestral archosaur genome at nucleotide level using highly fragmented assemblies from three crocodilian species (alligator, crocodile, and gharial), with the highest scaffold N50 being 508 Kb (in the case of the alligator). The draft reconstructed ancestral genome was, however, of a limited size (584 Mb) and aligned to only 26% of the alligator sequence. Moreover, the small median

scaffold size (and large number of scaffolds) meant that chromosome-level assembly was not currently possible and thus they were not amenable to analysis for our purposes. In any event, the wholesale fusion of all former microchromosomes[16] would presumably have made interpretation difficult.

Supplementary Note 3: Limitations of the bioinformatics (GRIMM, MGRA, MGRA2) analysis

In an ideal world, we would expect a 100% correspondence between molecular and bioinformatic analysis. However, the GRIMM tool uses the inputs of virtual data, which, in turn, are produced from whole genome sequence alignments using other computer programs/browsers and certain algorithms. The latter, in our case, was the genome browser Evolution Highway, and it was based on alignments of avian whole genome sequences against the chicken sequence in order to compare bird genomes.

In terms of what could compromise sequence alignments and subsequent identification of chromosomal changes in this in silico analysis: The alignment is dependent on the quality of both sequence and assembly of the compared genomes, on the one hand, and on the perfection of the algorithms used for this purpose, on the other. If the sequences are incomplete and poorly assembled, i.e. there are sequence gaps and genome assembly does not actually reflect the real genome organisation — and this is the case especially in birds, this may result in incorrect and incomplete sequence alignments of the compared genomes, and we may ultimately face identification of a reduced number of chromosome rearrangements. For this reason, we kept our analysis focussed on the best chromosomally assembled genomes (3 bird, 1 lizard, 1 mammal). MGRA2 particularly is limited in its ability to analyse sub-chromosomal fragmented genomes (like snakes, crocodiles and turtles). During the course of our analysis – we tried all of these without success.

FISH allows visualisation of the physical organisation of genomes as a set of chromosomes, and permits mapping of the chromosomal changes involved in the evolution of particular species. Yet, in the case of bird genomes, FISH is limited in its ability to identify intrachromosomal rearrangement in the microchromosomes but is more reliable in terms of showing interchromosomal changes.

As they are based on mathematical algorithms, there is no perfect reconstruction tool. The outcome of any computer program would depend on many factors particularly the input information that from one dataset to the other. For this reason, our results represent what we believe to be the “best

fit” by combining the molecular cytogenetic and bioinformatic data. Based on our experience however, two assumptions are reasonable 1) the number of CARs we identified was greater than the actual number of chromosomes in the DCA (this is discussed in relation to Figure 2); 2) there were more interchromosomal rearrangements that we did not identify.

Supplementary References

1. Benton, M. J. *et al.* Constraints on the timescale of animal evolutionary history. *Palaeontol. Electron.* **18,** 1–106 (2015).
2. Benton, M. J. & Twitchett, R. J. How to kill (almost) all life: the end-Permian extinction event. *Trends Ecol. Evol.* **18,** 358–365 (2003).
3. Nesbitt, S. J. *et al.* The earliest bird-line archosaurs and the assembly of the dinosaur body plan. *Nature* **544,** 484–487 (2017).
4. Sereno, P. C. The Evolution of Dinosaurs. *Science (80-.).* **284,** (1999).
5. Brusatte, S. L., Benton, M. J., Ruta, M. & Lloyd, G. T. The first 50Myr of dinosaur evolution: macroevolutionary pattern and morphological disparity. *Biol. Lett.* **4,** 733–6 (2008).
6. Jetz, W., Thomas, G. H., Joy, J. B., Hartmann, K. & Mooers, A. O. The global diversity of birds in space and time. *Nature* **491,** 444–448 (2012).
7. Griffin, D. K., Robertson, L. B. W., Tempest, H. G. & Skinner, B. M. The evolution of the avian genome as revealed by comparative molecular cytogenetics. *Cytogenet. Genome Res.* **117,** 64–77 (2007).
8. Cohen, M. M. & Gans, C. The chromosomes of the order Crocodilia. *Cytogenet. Genome Res.* **9,** 81–105 (1970).
9. Matsuda, Y. *et al.* Highly conserved linkage homology between birds and turtles: bird and turtle chromosomes are precise counterparts of each other. *Chromosom. Res.* **13,** 601–615 (2005).
10. Hillier, L. W. *et al.* Sequence and comparative analysis of the chicken genome provide unique perspectives on vertebrate evolution. *Nature* **432,** 695–716 (2004).
11. Warren, W. C. *et al.* The genome of a songbird. *Nature* **464,** 757–762 (2010).
12. Dalloul, R. A. *et al.* Multi-platform next-generation sequencing of the domestic turkey (Meleagris gallopavo): genome assembly and analysis. *PLoS Biol.* **8,** e1000475 (2010).
13. Rao, M. *et al.* A duck RH panel and its potential for assisting NGS genome assembly. *BMC Genomics* **13,** 513 (2012).
14. Zhang, G. *et al.* Comparative genomics reveals insights into avian genome evolution and

adaptation. *Science (80-.).* **346,** 1311–1320 (2014).

15. Green, R. E. *et al.* Three crocodilian genomes reveal ancestral patterns of evolution among archosaurs. *Science (80-.).* **346,** (2014).

16. Srikulnath, K., Thapana, W. & Muangmai, N. Role of Chromosome Changes in *Crocodylus* Evolution and Diversity. *Genomics Inform.* **13,** 102 (2015).
