# Supplementary material for: Reconstruction of the diapsid ancestral genome permits chromosome evolution tracing in avian and non-avian dinosaurs
Source: Nat Commun. 2018 May 21;9:1883. doi: 10.1038/s41467-018-04267-9 (PMC5962605; doi:10.1038/s41467-018-04267-9)
Supplement: Supplementary file 2 — Description of Additional Supplementary Files [file 41467_2018_4267_MOESM2_ESM.pdf]

Description of Additional Supplementary Files

File Name: Supplementary Data 1
Description: Size in base pairs (bp) of each DCA CAR.

File Name: Supplementary Data 2
Description: List and coordinates of each HBS/EBR identified. The ones highlighted in blue are those that were identified in the MGRA analysis (see Figure 2 and Supplementary Figure 1) as being involved in the inversions. Start and end coordinated refer to the chicken genome, equivalent chromosomes of duck, zebra finch, lizard and opossum are also given

File Name: Supplementary Data 3
Description: Cross species FISH results. Unless otherwise stated, results refer to chicken probes (chromosome paints or BACs) applied to the chromosomes of A. spinifera. Taken together, the data supports our central finding that the basic 'avian like' pattern was laid down in the archelosaur ancestor with few inter-chromosomal rearrangements since.

File Name: Supplementary Data 4
Description: Genes relevant to amino acid transmembrane transport (symport) and identified as having significant enrichments in the HSBs of the typical avian karyotype (with functionality adapted from UniProtKB; http://www.uniprot.org/uniprot/).

File Name: Supplementary Data 5
Description: Genes relevant to chromatin modification/organisation and identified as having significant enrichments in the EBRs of thetTypical avian karyotype (with functionality adapted from UniProtKB; http://www.uniprot.org/uniprot/)
